# Modelling hotspots of the two dominant Rift Valley fever vectors (*Aedes vexans* and *Culex poicilipes*) in Barkédji, Sénégal

**DOI:** 10.1186/s13071-016-1399-3

**Published:** 2016-02-27

**Authors:** Cheikh Talla, Diawo Diallo, Ibrahima Dia, Yamar Ba, Jacques-André Ndione, Andrew P. Morse, Aliou Diop, Mawlouth Diallo

**Affiliations:** Unité d’Entomologie Médicale, Institut Pasteur de Dakar, B.P. 220 Dakar, Sénégal; Laboratoire d’Etudes et de Recherches en Statistiques et Développement, Université Gaston Berger, Saint-Louis, Sénégal; Centre de Suivi Ecologique, Dakar, Sénégal; School of Environmental Sciences, University of Liverpool, Liverpool, UK; National Health Service, National Institute of Health Research, Health Protection Research Unit in Emerging and Zoonotic Infections, Liverpool, UK

**Keywords:** Rift Valley Fever, Mixed effects model, Logistic model, Spatial distribution model, Hotspot, Mosquitoes, Barkédji

## Abstract

**Background:**

Climatic and environmental variables were used successfully by using models to predict Rift Valley fever (RVF) virus outbreaks in East Africa. However, these models are not replicable in the West African context due to a likely difference of the dynamic of the virus emergence. For these reasons specific models mainly oriented to the risk mapping have been developed. Hence, the areas of high vector pressure or virus activity are commonly predicted. However, the factors impacting their occurrence are poorly investigated and still unknown. In this study, we examine the impact of climate and environmental factors on the likelihood of occurrence of the two main vectors of RVF in West Africa *(Aedes vexans* and *Culex poicilipes)* hotspots.

**Methods:**

We used generalized linear mixed models taking into account spatial autocorrelation, in order to overcome the default threshold for areas with high mosquito abundance identified by these models. Getis’ *G*i*(*d*) index was used to define local adult mosquito abundance clusters (hotspot).

**Results:**

For *Culex poicilipes*, a decrease of the minimum temperature promotes the occurrence of hotspots, whereas, for *Aedes vexans*, the likelihood of hotspot occurrence is negatively correlated with relative humidity, maximum and minimum temperatures. However, for the two vectors, proximity to ponds would increase the risk of being in an hotspot area.

**Conclusions:**

These results may be useful in the improvement of RVF monitoring and vector control management in the Barkedji area.

**Electronic supplementary material:**

The online version of this article (doi:10.1186/s13071-016-1399-3) contains supplementary material, which is available to authorized users.

## Background

Rift Valley fever (RVF) is an emerging arboviral disease considered as a threat to human and animal health in Africa and Arabian Peninsula. RVF outbreaks have led to significant economic impact [1, 2]. This disease affects mainly cattle, sheep, and goats and is responsible for abortion among pregnant females and increasing new-born mortality [3, 4]. In humans, RVF infections are usually asymptomatic, but severe cases can result in complications including haemorrhagic syndromes which are associated with high mortality rates [5]. Humans are exposed to the Rift Valley fever virus (RVFV) by direct contact with infected animals, organs of dead animals, fluids, aerosols, raw milk, and bites of infected mosquitoes [6, 7]. RVFV is transmitted by several species of mosquitoes, mainly of the genera *Aedes* and *Culex*. In Senegal, after the RVF outbreak in Mauritania in 1987, an entomological and animal surveillance program was implemented in several biogeographic zones [8–10]. These studies highlighted several RVFV circulation. Thus, the RVFV was isolated from several mosquito species collected at the end of the rainy season in the Barkedji area [8, 11]. In this area, six mosquito species were found associated to RVFV but the two dominant vectors are *Culex poicilipes* and *Aedes vexans* [12–14]. All these vectors breed in temporary ponds that are flooded after the first rains usually in July. These temporary ponds represent the main source of water for people, livestock and wildlife in this area for at least 6 months per year [14, 15].

The impact of environmental and climatic factors on the heterogeneous spatial distribution of several species of mosquitoes is complex and poorly understood despite the valuable models developed in the recent years [16–19]. This is specially the case for vector-borne diseases such as RVF where knowledge of the relation between host, vectors, and environmental factors is useful for understanding the persistence, emergence, transmission and possible amplification of the virus.

The active circulation of the RVFV underlines the need for efficient surveillance and control tools. To assist prudent decision making in animal and human health resource allocation for surveillance and control, spatio-temporal models for outbreaks prediction have been developed using climate time series data such as rainfall and sea surface temperature and the Normalized Difference of Vegetation Index (NDVI) anomalies map to set up an early warning system for East Africa and Arabian Peninsula [20–22]. However, this system has not been effective in West Africa because the factors involved in the emergence and re-emergence of this virus appear to be different [17]. These included the lack of sensitivity of the system like threshold of NDVI and mosquito vectors involved in the transmission [23]. Further, in contrast in East Africa, above normal rainfall is not always associated to RVFV emergence in West Africa [24]. Thus, for West Africa, several studies have been conducted particularly in Senegal to generate an early warning system [18, 25–28]. One of the main approaches was to focus on the abundance of the main vectors involved in RVFV transmission. Precisely at Barkedji, a Bayesian model was implemented using Markov chain Monte Carlo (MCMC) methods [29]. The main purpose of this previous modelling approach was to define areas of high and low vector abundance, in order to first identify appropriate places for pastoralists to settle and then to minimise contact between vectors and hosts. The other objective was to provide information to the authorities for the implementation and adaptation of targeted vector control strategies based upon small-scale insecticide use after identification of areas to be treated. This statistical model was used to assess the effects of climatic and ecological determinants on the spatiotemporal dynamics of *Ae. vexans* and *Cx. poicilipes* at a local-scale. However, due to the lack of abundance threshold, the previous model developed does not allow to rank among high density areas. This ranking is necessary to reduce costs and surfaces to be treated but also manage the problem of vector resistance to insecticides and the issue of environmental pollution. Indeed, previous studies using satellite images in the Barkédji area have identified during the 2003 rainy season a total of 468 ponds in July [30] and 1354 ponds in August [31]. This large number of ponds demonstrates the need of classification tools in the presence of several sites with high mosquito densities to determine those of primary importance. This is especially important for low income countries where resources are scarce and appropriate guidance is essential for preventing mosquito bites or reducing vector abundance. For that purpose, we developed, in this study, a model to determine the impact of climate and environmental data on the occurrence of hotspots. This model would be an alternative to the lack of threshold. Thus, sites with high densities, predicted by the previous model [29] and with a high probability of being hotspots will be considered in priority as risk areas for any intervention program. We initially determined all hotspots of the two main vectors in our study area using Getis-Ord statistic.

The Getis–Ord Gi* hotspot cluster statistic is one of the possible approaches that can be used for local spatial analysis [32]. This statistics identifies those clusters with higher values (hotspot) in magnitude than expected to be found by random chance. The Gi* statistic measures the degree of spatial clustering of a local sample and indicates how different it is from the expected value which is the mean of the whole dataset. It was used to determine the distribution of multiple mosquito species and to show how they may currently and in the future be affected by climate change, control interventions or other factors [33–35]. It can also be used to help decision maker in identifying areas at risk to target for strategies such as spraying and larval control [36–39].

Statistical species distribution models, including generalized linear models, are commonly used as a tool in decision-making [40–42]. These models also commonly use environmental and climate factors as explanatory variables but their spatial variation influencing predicted distributions are rarely quantified. Hence, the knowledge of climatic and environmental factors affecting the occurrence of these hotspots is required as information-support for decision-making.

The aims of this study were to identify local adult *Ae. vexans* and *Cx. poicilipes* abundance clusters (hotspots) using the Getis’ *G*i*(*d*) index and to model the occurrence of these hotspots by generalized linear mixed models (GLMM). We adopted this modelling approach to quantify the effect of cumulative rainfall, relative humidity, maximum temperature, minimum temperature and the NDVI and the Euclidian distance to larval breeding sites (temporary ponds) on the distribution of vector hotspots. This approach could assist in identifying areas at higher risk and further provide important information supporting decision making including how different intervention actions should be spatially allocated.

## Methods

### Study area

**Fig. 1:**
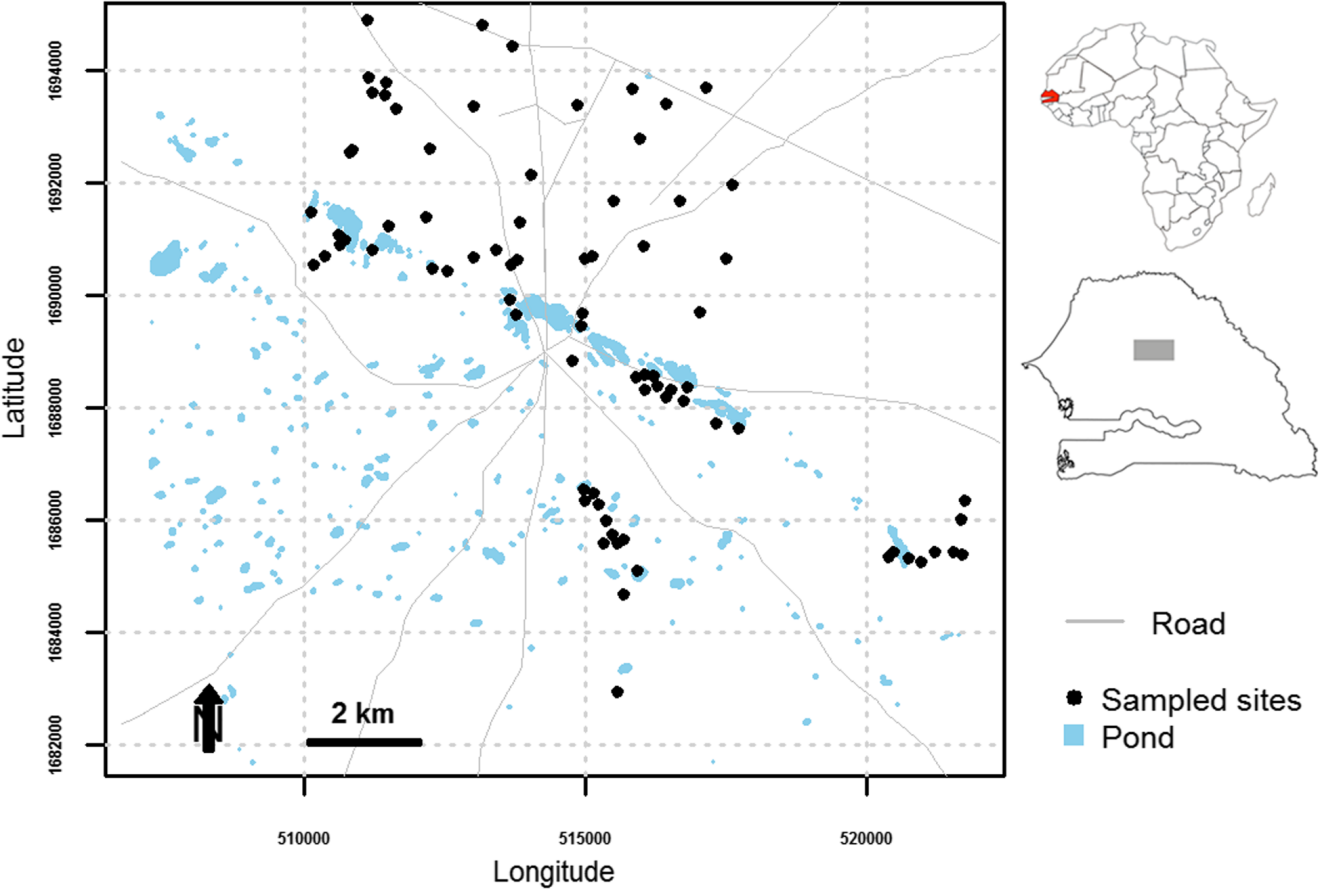
Study area

The study was performed around the Barkedji village (14°52’02”W, 15°16’41”N), in the Ferlo area (central north of Senegal) during the 2005 to 2006 rainy seasons (Fig. 1). This area is characterised by a hot dry climate, short rainy season (from June to October), and long dry season (November to May), with annual rainfall ranging from 300 to 500 mm and a number of rainy days around 35.8 [17]. The highest temperatures are usually realised during October with average daily maximum of 45 °C and the lowest temperatures during December with 16 °C. Many temporary ponds flood at the beginning of the rainy season and represent the main source of water for people and livestock. These ponds are the natural habitats of many species of birds, reptiles and rodents and the oviposition and resting sites for mosquito vectors of RVFV. Cattle farming is the main activity of the people in this area with other livestock mainly sheep and goats. The houses are scattered around temporary ponds and are usually composed of small villages with huts.

### Mosquito population sampling and explanatory variables

Mosquitoes were sampled from 79 sites once every 2 weeks from July to December 2005 and 2006 by CO_2_-CDC light traps (model 512; John W. Hock Company, Gainesville, FL) for a period of 12 h (6 pm to 6 am). Upon collection, mosquitoes were euthanised and identified using available morphological keys [43, 44]. The geographical coordinates of each site were recorded with a Garmin GPSMAP 76S (Olathe, KS, USA) hand-held global positioning unit and projected in UTM Zone 28 N allowing the Euclidian distances from each site to the nearest pond to be estimated using a spatial map of the region.

Rainfall, relative humidity, maximum and minimum temperature, NDVI and distance from the nearest pond were the variables selected to model vector hotspots occurrence. The meteorological variables were collected from automatic weather stations (BWS 200 Campbell Scientific) installed in the Barkedji area. Rainfall represented the cumulative rainfall 15–20 days prior to trapping. We calculated the averages for maximum and minimum temperature, and relative humidity of the sampling period. Remotely-sensed Moderate resolution Imaging Spectroradiometer (MODIS) data set were sourced from the National Aeronautic and Spatial Administration (http://neo.sci.gsfc.nasa.gov/view.php?datasetId=MOD13A2_E_NDVI.) and the NDVI with spatial and temporal resolution of 250 m and 16 days intervals analysed. These explanatory variables chosen are considered likely to be important determinants of the two vectors distribution.

Clustered localities with high or low *Ae. vexans* and *Cx. poicilipes* abundances were identified using the Getis-ord Gi* statistic [32] in R software with “spdep” package [45]. Statistically significant (at a level of 0.05) clusters of sites with high vector abundances were identified as hotspots with Z scores > 1.96, while Z scores of < -1.96 represented coldspots with low vector abundance. The spatial relationship among sites was conceptualised using the inverse distance, which is most appropriate for continuous point datasets because closer sites have larger influences on the computation for each target site than sites that are further away. Data on presence/absence of hotspot was coded as binary factor (presence =1 or absence = 0). At each site, the presence or absence of hotspot (Z scores > 1.96 for presence and Z scores < 1.96 for absence) were then determined.

### Statistical modelling

The generalized linear mixed model (GLMM) with a binomial distribution was fitted taking into account spatial autocorrelation with the Getis-Ord Gi* Z score as the dependent factor. We modelled the likelihood of the presence of hotspot by introducing a random intercept effect between sites [46]. The general form of the logistic regression models is:

$$ ln\left(\frac{p_{st}}{1-{p}_{st}}\right) = {\beta}^{\hbox{'}}{X}_{st} + {b}_s $$

Where *p*_*st*_ is the probability of vector hotspot presence at site s at fortnight t, *β* is a vector of coefficient; *X*_st_ is a vector of covariates for site s at time t; and *b*_s_ is the random effect for site s. The parameter *b*_s_ is distributed normally with mean zero and variance *σ*^2^(*b*_*s*_ ~ *Normal*(0, *σ*^2^)). GLMM is an alternative for taking into account the spatial autocorrelation within sites [47]. The R package “glmmML” [48] and previously used R source codes were modified and utilised for model fitting [47]. For the GLMM model, we used the methodology described [47].

To verify possible spatial autocorrelation in the parsimonious model residuals, Moran’s I correlograms plot (with a lag interval of 5000 m) from the Pearson residuals of the generalized linear model (GLM) and the GLMM was used [49, 50].

### Model selection

The goodness of fit was assessed for all models tested using the Akaike information criterion (AIC) criteria [51]. AIC criteria is define as:

$$ AIC=-2L+2K $$

where L is the maximum log-likelihood of the model and K is the number of parameters in the model. The best approximating model is the model with the lowest AIC.

We tested different combinations of the explanatory variables in the model. Each model were fitted and ranked by their AIC values. We also used Akaike weights *w*_*i*_ to calculate the relative probability of each model compared to the best model. The Akaike weights for model *i* is defined as [52]:

$$ {w}_i = \frac{exp\left(-\frac{1}{2}{\Delta}_i\right)}{{\displaystyle {\sum}_j^R} exp\left(-\frac{1}{2}{\Delta}_j\right)} $$

Where ∆_*i*_ is the difference between the AIC for model *i* and the best model (with the lowest AIC) and the sum in the denominator is over all candidate models (*j* = 1,…,*R*), the denominator is the sum of the relative likelihoods for all candidate models. Finally, for each covariate, the relative importance was evaluated by an indicator obtained by summing the Akaike weights for all models containing the covariate [52]. A 95 % confidence set of models was obtained by ranking model by their Akaike weights and added them successively until the sum exceeds 0.95. We used the area under receiver operating curve (ROC) for the best model to assess its predictive capacity [53, 54]. Values of area under a ROC curve (AUC) greater than 0.7 indicate that the predictive capacity of model may be considered acceptable, excellent for AUC ≥ 0.9, and poor if AUC value is lower than 0.6 [55]. ROC curve were done using ROCR package [56] with the R software [57].

We used graphical methods, quantile-quantile plots with simulation approach and partial residuals plots, to assess the goodness of model fit [58]. Then we applied these methods to the most parsimonious model. The partial residual graph is a plot of the values of a specific covariate in the model versus its partial residuals. Partial residuals *r*_*par*_ are defined as:

$$ {r}_{par}=\frac{y-\widehat{p}}{\widehat{p}\left(1-\widehat{p}\right)}+X{\widehat{\beta}}_X $$

Where y is the observed data (1 or 0), $\widehat{p}$ is probability estimated by the fitted model, *X* is the model covariate, and ${\widehat{\beta}}_X$ is the estimated coefficient for the covariate *X* [58]. A linear partial residual plot indicates that linear assumption in the model is adequate. However, a non-linear of partial residual plot suggests that model linear assumption may be not suitable [47]. The partial residual plots were only done for covariates in the most parsimonious model with smoothed curves fitted.

To improve predictive performance of the model, we applied a model averaging method to take into account model uncertainty [52]. Model averaged predictions are more robust than those derived from a single model [47]. Model coefficients were estimated and predictions made taking into account parameter uncertainty [52].

## Results

**Fig. 2:**
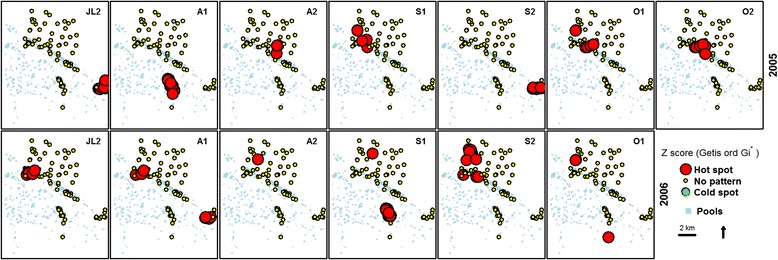
Spatial clustering of *Aedes vexans* abundances over each fortnight in 2005 and 2006 during the rainy season. The red color represents the hotspot of abundance (Z score of the Getis-Ord >1.96; statistically significant), the green color the cold spot of abundance (Zscore of the Getis-Ord < - 1.96; statistically significant) and the yellow color no pattern (Z score of Getis-Ord between -1.96 and 1.96; not statistically significant). JL2 represents the second fortnight of July and A1 the first fortnight of August

**Fig. 3:**
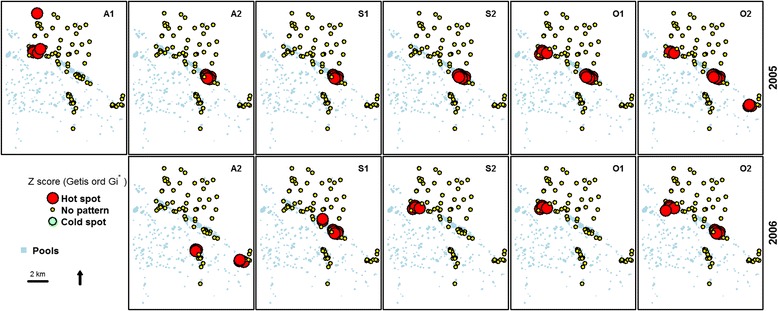
Spatial clustering of *Culex poicilipes* abundances over each fortnight in 2005 and 2006 during the rainy season. The red color represents the hotspot of abundance (Z score of the Getis-Ord >1.96; statistically significant), the green color the cold spotof abundance (Zscore of the Getis-Ord < - 1.96; statistically significant) and the yellow color no pattern (Z score of Getis-Ord between -1.96 and 1.96; not statistically significant)

The localities of high and low clusters of vectors abundances were identified with the Z scores computed by Getis-Ord Gi* (Figs. 2 and 3). For *Aedes vexans*, there were 35 hotspots sites in 2005 and 32 hotspots sites in 2006. For *Culex poicilipes*, there were 19 hotspots sites in 2005 and 21 hotspots sites in 2006 (Table 1). For *Aedes vexans*, in 2005, the first half of August had the highest hotspots numbers (11) whereas in 2006 it was during the second half of September (11). In 2005, *Culex poicilipes* had 15 hotspots in the second half of October and 12 hotspots in 2006 (Table 1).

**Table 1:** Number of hotspots (presence of hotspot among 70 collected sites) for each fortnight during the study period

Vector	Year	JL2	A1	A2	S1	S2	O1	O2	Number of hotspots
*Aedes vexans*	2005	7	11	3	5	7	6	8	35
	2006	6	8	1	10	11	3	0	32
*Culex poicilipes*	2005	0	6	6	6	9	14	15	19
	2006	0	0	7	8	5	5	12	21

JL2: represents the second fortnight of July. Clusters of sites with high abundances of mosquito were identified with Z score > 1.96

The most parsimonious model for *Cx. poicilipes* (AIC = 416.6) contained the distance to the nearest pond and the minimum temperature (Tables 2 and 3). The standard deviation of random-effects estimated was 3.07 ± 0.93 indicating relatively large variation amont collection sites.

**Table 2:** Coefficient estimates for the 95 % confidence set of models and the model-average, the relative importance indices and AIC for *Aedes vexans*

Model ranking	Intercept	Distance	Hr	NDVI	Rainfall	Tmax	Tmin	AIC	weight
1	21.60	−0.0017	−0.0618			−0.2508	−0.4157	571.3	0.368
2	22.02	−0.0017	−0.0723	1.988		−0.2556	−0.4231	571.8	0.283
3	21.77	−0.0018	−0.0671		0.0019	−0.2382	−0.4325	572.9	0.168
4	22.03	−0.0017	−0.074	1.830	0.0009	−0.2487	−0.4297	573.7	0.109
Relative Importance		0.958	0.975	0.419	0.299	0.994	0.992		
Model-average	21.810 ± 11.19^a^	−0.002 ± 0.001^a^	−0.067 ± 0.043^a^	1.944 ± 3.329	−0.001 ± 0.006	−0.250 ± 0.130^a^	−0.423 ± 0.225^a^		

^a^indicates significance at the 95 % level. *Hr* the relative humidity, *Tmax* maximum temperature, *Tmin* minimum temperature; Rainfall: cumulative rainfall

**Table 3:** Coefficient estimates for the 95 % confidence set of models, the model-average, and the relative importance indices, AIC for *Culex poicilipes*

Model ranking	Intercept	Distance	Hr	NDVI	Rainfall	Tmax	Tmin	AIC	weight
1	4.989	−0.0053					−0.3559	416.6	0.155
2	4.169	−0.0053			−0.0038		−0.3106	417.0	0.123
3	4.967	−0.0053		−2.5660			−0.3159	417.2	0.116
4	7.692	−0.0053			−0.0058	−0.0641	−0.3561	418.2	0.067
5	5.018	−0.0053	−0.0060				−0.3386	418.4	0.062
6	4.986	−0.0053				0.0001	−0.3559	418.6	0.057
7	4.423	−0.0053		−1.5370	−0.0025		−0.3015	418.7	0.053
8	4.116	−0.0053	0.0023		−0.0040		−0.3150	419.0	0.046
9	4.953	−0.0053	0.0039	−2.8640			−0.3228	419.1	0.044
10	5.648	−0.0053		−2.6260		−0.0111	−0.3268	419.1	0.043
11	7.448	−0.0053		−0.9438	−0.0048	−0.0569	−0.3452	420.1	0.026
12	7.714	−0.0053	0.0039		−0.0062	−0.0660	−0.3652	420.2	0.026
13	5.292	−0.0053	−0.0062			−0.0044	−0.3428	420.4	0.023
14	4.341	−0.0053	0.0065	−1.9120	−0.0028		−0.3115	420.6	0.021
Relative importance		0.898	0.280	0.373	0.421	0.307	0.991		
Model-average	5.180 ± 6.21	−0.005 ± 0.01^a^	0.000 ± 0.03	−2.251 ± 4.92	−0.004 ± 0.01	−0.033 ± 0.14	−0.333 ± 0.20^a^		

^a^indicates significance at the 95 % level. *Hr* the relative humidity, *Tmax* maximum temperature, *Tmin* minimum temperature, Rainfall: cumulative rainfall

There was negative correlation between distance to nearest waterpond, minimum temperature and vector hotspot occurrence. However, this model was just 1.26 times better than the model incoporating cumulative rainfall (evidence ratio = 0.155/0.123) and (0.155/0.116) and 1.34 times better than the model with NDVI, distance to the nearest pond and the minimum temperature parameters. The best model for *Ae. vexans* (AIC = 571.3) contained distance to the nearest pond, minimum and maximum temperature and relative humidity (Tables 2 and 3). The standard deviation of random-effects estimated was 0.37 ± 0.27 indicating weak variation among collection sites. These variables were all negatively correlated with this vector hotspot occurrence. Nevertheless, this model was 1.3 (0.368/0.283) times better than the model containing NDVI variable and 2.2 (0.368/0.168) times better than the model with cumulative rainfall.

**Fig. 4:**
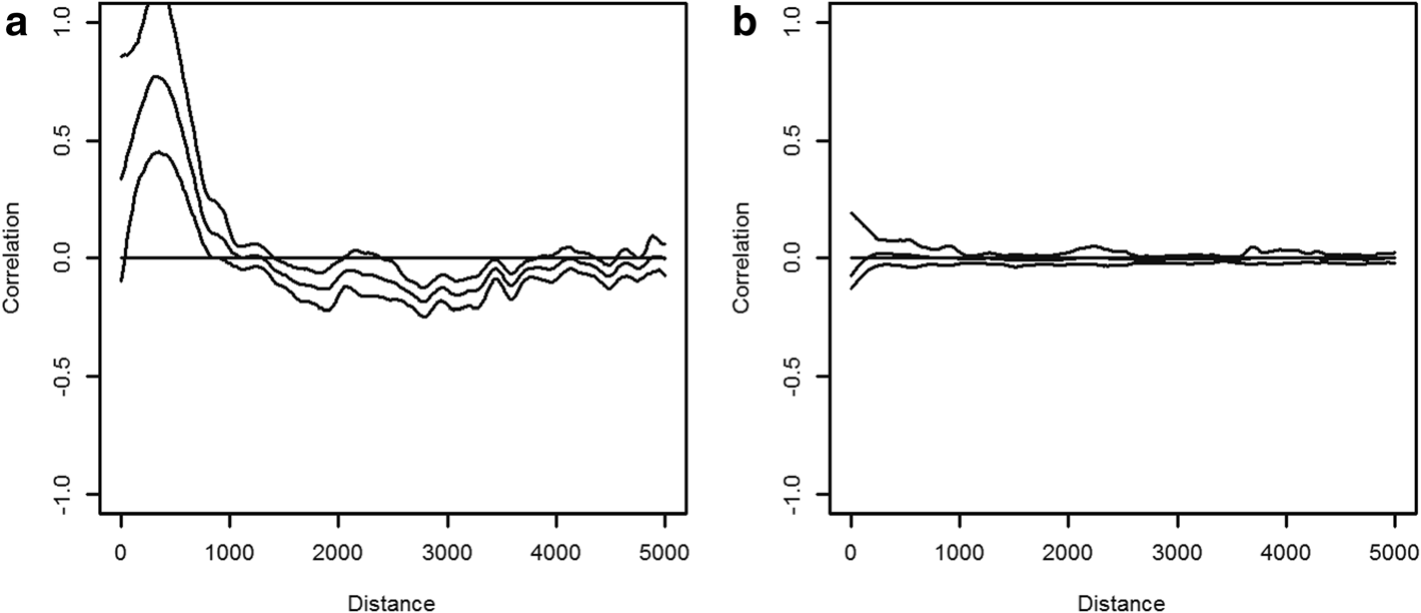
Spline correlograms with 95 % pointwise bootstrap confidence intervals; the Pearson residuals from the parsimonious model without mixed effects (**a**) and with mixed effects (**b**) for *Cx. poicilipes*

**Fig. 5:**
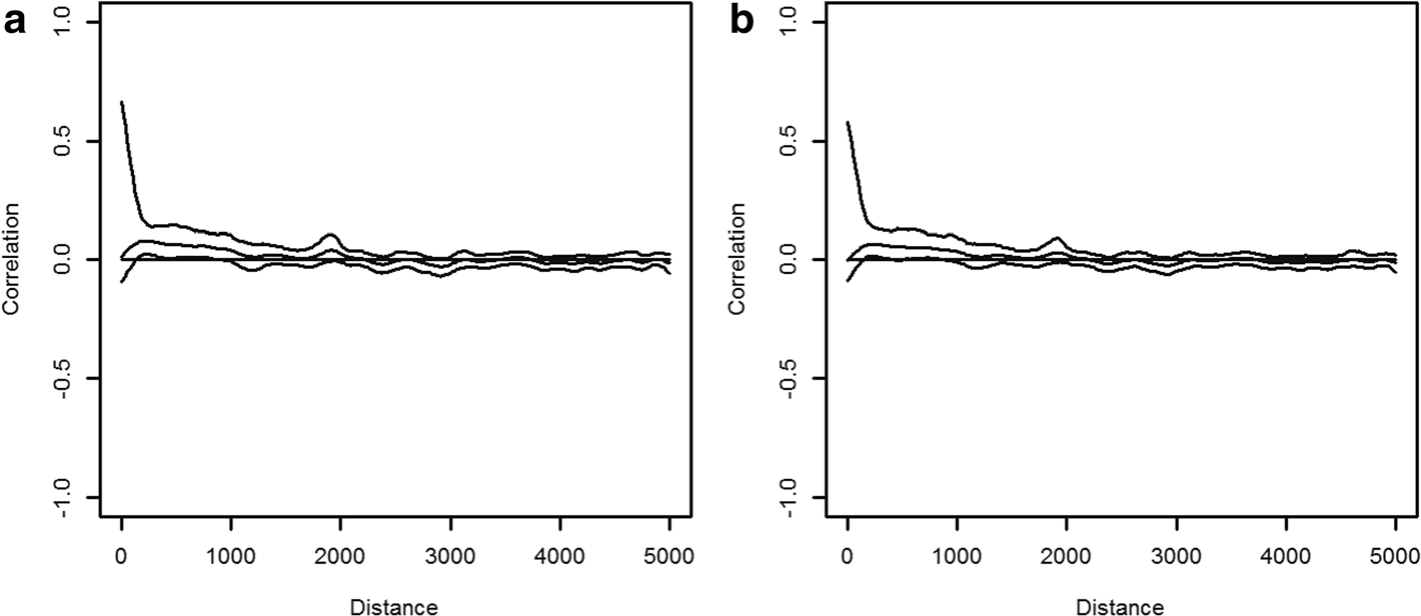
Spline correlograms with 95 % pointwise bootstrap confidence intervals; the Pearson residuals from the parsimonious model without mixed effects (**a**) and with mixed effects (**b**) for *Ae. vexans*

Significant positive spatial autocorrelation was observed in the model residuals (Fig. 4), but at short lag distances of less than 1.0 km for *Cx. poicilipes*. This indicates that spatial autocorrelation is a problem for sites located close to each other. Observations from these sites can not be considered as independent. However, the spline correlogram plot of the Pearson residuals generated by the GLMM model (Fig. 4) showed absence of spatial correlation. This indicates that the mixed model successfully takes into account the spatial autocorrelation existing within sites. Concerning *Ae. vexans*, the spline correlogram plot demonstrated absence of spatial autocorrelation in Pearson residuals of GLM and GLMM models (Fig. 5).

All graphical methods used to test the goodness of model fit for both species indicated no considerable deviation from the models hypothesis. The first one was the quantile-quantile plots which points were inside the simulated 95 % confidence interval (Additional file 1: Figure S1-A). Partial residuals plots, the second one, with smoothed curve, indicated that the linear hypothesis for each covariate appeared to be suitable (Additional file 2: Figure S1-B and Additional file 3: Figure S1-C).

**Fig. 6:**
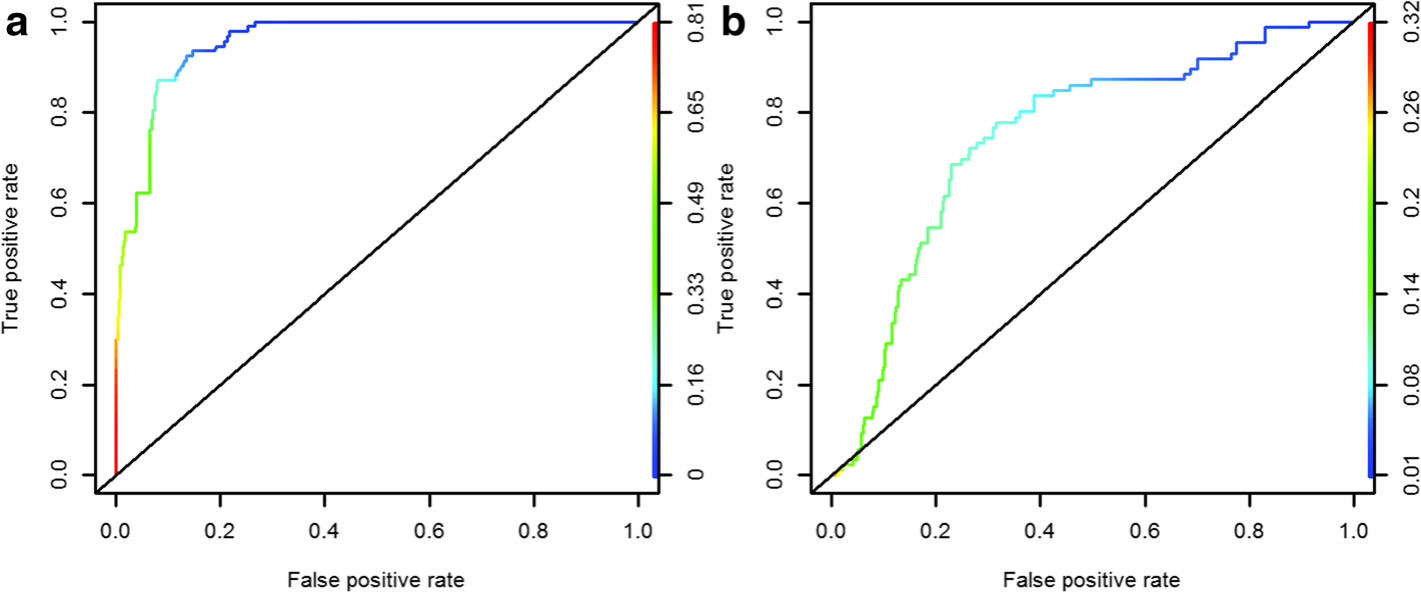
ROC curve of vector hotspot occurrence; **a**: for *Cx. poicilipes* (AUC = 0.96) and **b**: for *Ae. vexans* (AUC = 0.75)

The cross-validation area under the curve (AUC) was 0.96 and 0.75 for *Cx. poicilipes* and *Ae. vexans,* respectively (Fig. 6), indicating stronger predictive ability and that the framework of the most parsimonious models for each species were appropriate.

For *Ae. vexans*, the most important variable that influenced abundance was maximum temperature (relative importance index = 0.994), followed by minimum temperature (0.992), relative humidity (0.975) and distance to the nearest pond (0.958) (Table 2).

For *Cx. poicilipes*, minimum temperature (0.991) and distance to the nearest pond (0.898) were the most mportant variables that influenced the occurrence of mosquito hotspots (Table 3).

## Discussion

Our study focused on *Cx. poicilipes* and *Ae. vexans* because they are the most abundant and are considered as the main vectors of RVF in the area. They also exhibit a high degree of interaction with the main vertebrate hosts of the virus [11, 14]. Other mosquito species like *Mansonia uniformis, Mansonia africana, Aedes fowleri, Aedes ochraceus*, were found associated with RVF virus. However, they are uncommon and accidently infected. *Mansonia uniformis* and *Ma. africana* are occasionally abundant but their association with aquatic vegetation limits their spatial distribution [59, 60]. Here we developed a statistical model of the RVF vectors hotspot distribution where the probability of hotspot presence was conceptualised as a function of climatic and environmental variables. Our approach demonstrated that climatic and environmental variables were very important for determining the distribution of RVF vectors hotspot in Barkedji area.

There was inverse relationship between distance to pond and predicted probability of hotspot areas for both species. The high abundance of the main RVF vectors near temporary ponds agreed with previous studies that estimated the average flying range less than 600 m and 650 m for *Cx. poicilipes* and *Ae. vexans,* respectively [24]. A study showed also that their abundances decrease linearly up to 843 and 1,394 m from a given pond for *Cx. poicilipes* and *Ae. vexans,* respectively [12]. In addition, *Cx. poicilipes* were generally found near some ponds located in the Ferlo riverbed [11, 14, 61]. The distance of a host to the ponds is an important factor that could affect the risk of being infected by RVFV. It may be an indicator to find the potential vertebrate reservoir host of the virus.

During this study, the lowest and highest temperature ranges recorded were 20.57 °C and 45.39 °C. The negative correlation between minimum temperature and occurrence of vector hotspots indicate that the vectors are very sensitive to lower temperatures that impact negatively on their survival and ability to transmit pathogens such as dengue virus [62, 63]. In addition, vector biology studies showed that higher transmission of RVFV was observed at higher temperatures for *Aedes* and for *Culex* [64–66]. However, the recent emergence of RVFV in Madagascar illustrates that RVFV is also able to circulate under more temperate condition [67, 68].

Previous studies in West Africa have shown that, from 1961 to 2003, periods of RVFV emergence did not coincide with years of high rainfall [16, 18] and that *Ae. vexans* and *Cx. poicilipes* abundance and total rainfall were not correlated [17]. In West Africa, intra seasonal variability of rainfall was suspected to have more impact on mosquitoes dynamics [69]. Therefore, the dynamics of filling ponds was recently identified as a favourable factor in the abundance of the two vectors [18]. RVF outbreaks in Tanzania during 2006–2007 was preceded with periods of above normal rainfall and the occurrence of outbreaks was associated with total amount of rainfall above a threshold (405.4 mm) [70].

The relative performance of several alternative models tested could arise because of correlations between environmental variables, which would consequently limit the amount of additional information derived from varying specific combinations between the variables tested. However, in the model with averaged coefficients, only the two factors (NDVI and cumulative rainfall) had a high likelihood of being included in the parsimonious model. These variables, although not statistically significant in the averaged model, had a great influence in predicting vector hotspot occurrence. Model-averaged could be used to predict the presence of hotspot. The concept of inference is to reduce model selection biais effects on linear regression coefficient estimates [52].

## Conclusions

Vector control is challenging in Barkedji area due to the heterogeneous spatial distribution of RVF vectors across landscape as well as their breeding habitat. The identification of vectors hotspots is a key step in the implementation of most efficient surveillance and control strategies. Thus, this monotoring should target hotspot sites. Pastoralists should be advised to avoid hotspots areas in order to reduce the probability of contact between host and vectors before the government could be able to make more safer and modern water sources for the herds in the future.

## Additional files

Additional file 1: Figure S1. Quantile-quantile plot with 95 % pointwise confidence bounds; (A) for *Ae. vexans* and (B) for *Cx. poicilipes*. (TIF 37 kb) Additional file 2: Figure S2. Partial residuals plots for covariates in the parsimonious model for *Cx. poicilipes*; (A) for distance, (B) for maximum temperature, (C) for minimum temperature and (D) for relative humidity. (TIFF 51 kb) Additional file 3: Figure S3. Partial residuals plots for covariates in the parsimonious model for *Ae. vexans*; (A) for distance and (B) for minimum temperature. (TIFF 25 kb)
