# Supplementary figures and images for: Modelling hotspots of the two dominant Rift Valley fever vectors (Aedes vexans and Culex poicilipes) in Barkédji, Sénégal
Source: Parasit Vectors. 2016 Feb 27;9:111. doi: 10.1186/s13071-016-1399-3 (PMC4769837; doi:10.1186/s13071-016-1399-3)

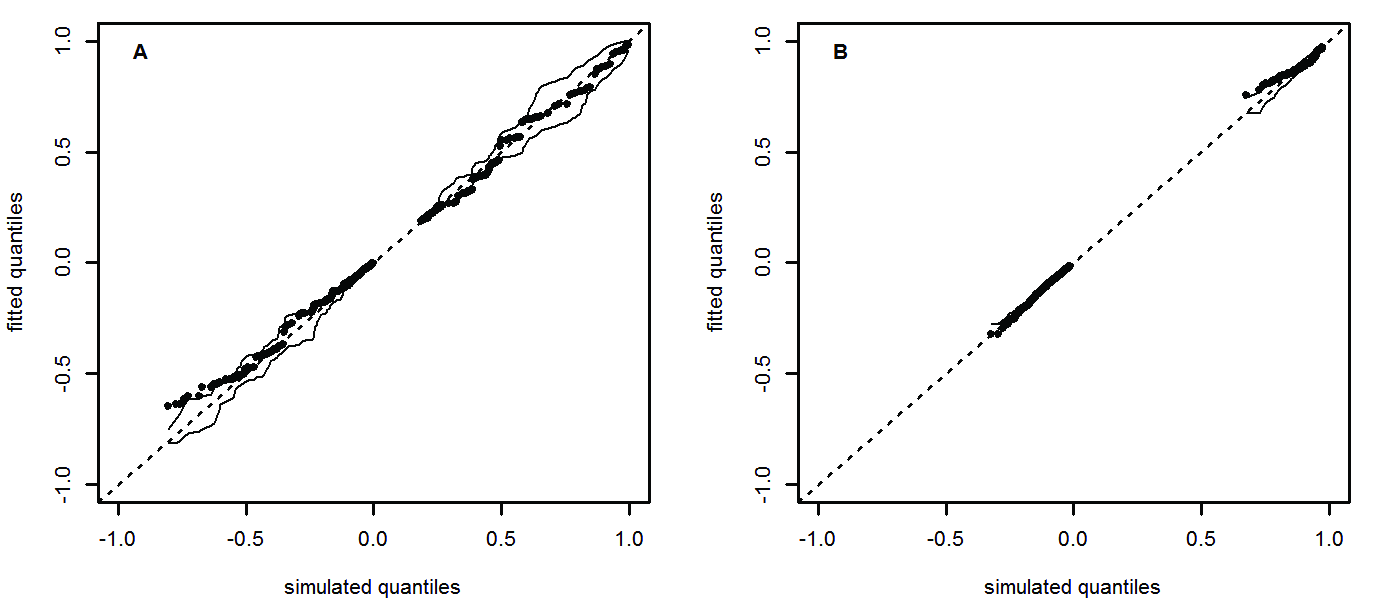

Supplement: Additional file 1: Figure S1. — Quantile-quantile plot with 95 % pointwise confidence bounds; (A) for Ae. vexans and (B) for Cx. poicilipes. (TIF 37 kb) [file 13071_2016_1399_MOESM1_ESM.tif]

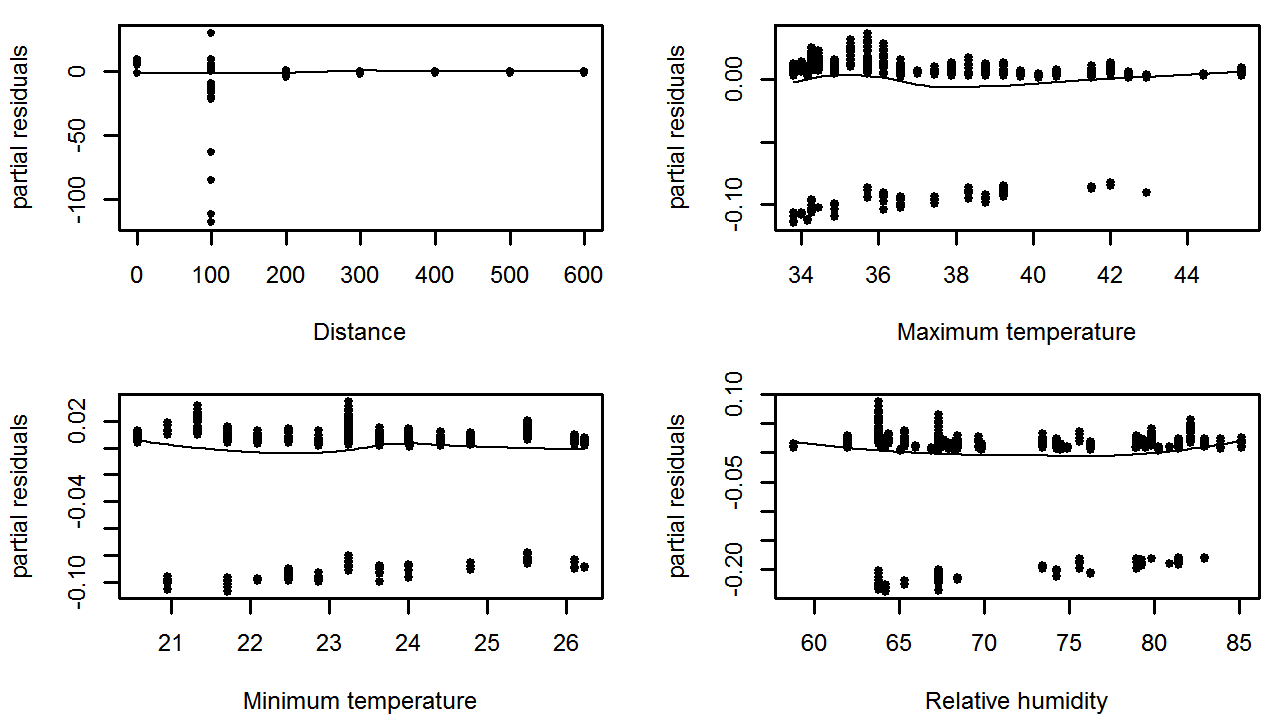

Supplement: Additional file 2: Figure S2. — Partial residuals plots for covariates in the parsimonious model for Cx. poicilipes; (A) for distance, (B) for maximum temperature, (C) for minimum temperature and (D) for relative humidity. (TIFF 51 kb) [file 13071_2016_1399_MOESM2_ESM.tiff]

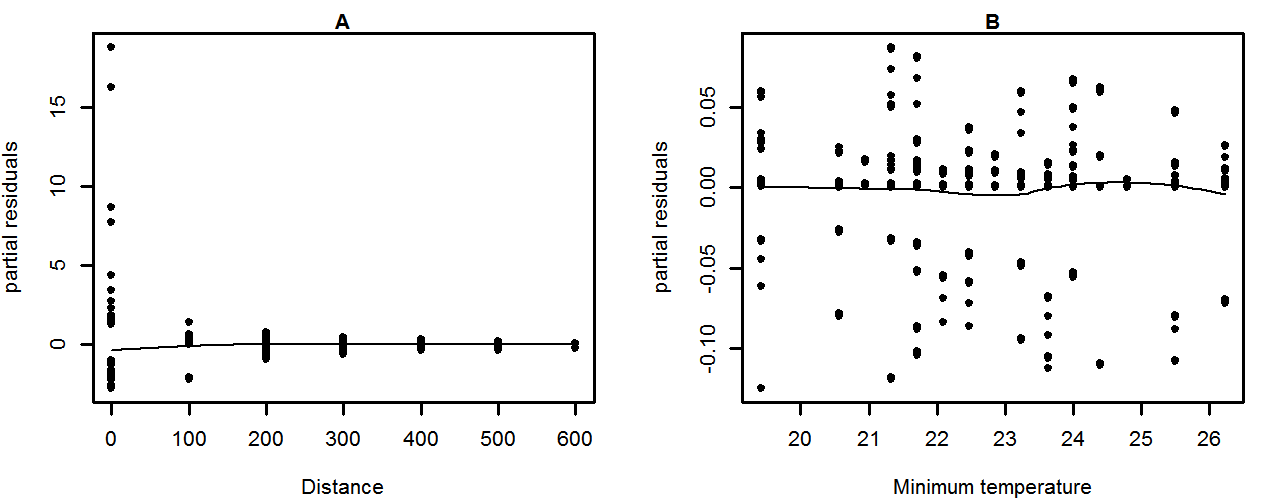

Supplement: Additional file 3: Figure S3. — Partial residuals plots for covariates in the parsimonious model for Ae. vexans; (A) for distance and (B) for minimum temperature. (TIFF 25 kb) [file 13071_2016_1399_MOESM3_ESM.tiff]
